# Apoptotic and Necroptotic Mediators are Differentially Expressed in Mucinous and Non-Mucinous Colorectal Cancer

**DOI:** 10.3389/fonc.2022.815001

**Published:** 2022-07-14

**Authors:** Emer O’Connell, Ian S. Reynolds, Andreas U. Lindner, Manuela Salvucci, Tony O’Grady, Orna Bacon, Sanghee Cho, Elizabeth McDonough, Daniel Longley, Fiona Ginty, Deborah A. McNamara, John P. Burke, Jochen H. M. Prehn

**Affiliations:** ^1^ Department of Physiology and Medical Physics, Royal College of Surgeons in Ireland, Dublin, Ireland; ^2^ Department of Colorectal Surgery, Beaumont Hospital, Dublin, Ireland; ^3^ Centre for Systems Medicine, Royal College of Surgeons in Ireland, Dublin, Ireland; ^4^ Department of Pathology, Beaumont Hospital, Dublin, Ireland; ^5^ GE Global Research, Niskayuna, NY, United States; ^6^ Centre for Cancer Research and Cell Biology, Queen’s University Belfast, Belfast, United Kingdom; ^7^ Department of Surgery, Royal College of Surgeons in Ireland, Dublin, Ireland

**Keywords:** apoptosis, necroptosis, mucinous, colorectal, chemotherapy

## Abstract

**Background:**

Mucinous colorectal cancer (CRC) represents 10% of all CRC and is associated with chemotherapy resistance. This study aimed to determine expression of apoptosis and necroptosis mediators in mucinous CRC.

**Methods:**

RNA gene expression data were extracted from TCGA. Protein levels in 14 mucinous and 39 non-mucinous tumors were measured by multiplexed immunofluorescence. Levels of apoptosis and necroptosis signalling proteins were analysed in SW1463 (mucinous rectal), SW837 (non-mucinous rectal), LS174T (mucinous colon) and HCT116 (non-mucinous colon) cell lines by western blot. Cell death was investigated by flow cytometry measurement of propidium iodide stained cells.

**Results:**

High cleaved-Caspase 3 expression was noted in resected mucinous tumors. Western blot identified alterations in apoptosis proteins in mucinous CRC, most prominently downregulation of Bcl-xL protein levels (p=0.029) which was also observed at the mRNA level in patients by analysis of TCGA gene expression data (p<0.001). Treatment with 5-FU did not significantly elevate cell death in mucinous cells, while non-mucinous cells showed robust cell death responses. However, 5-FU-induced phosphorylation of MLKL in mucinous cancer cells, suggestive of a switch to necroptotic cell death signaling.

**Conclusion:**

Apoptotic and necroptotic mediators are differentially expressed in mucinous and non-mucinous colorectal cancers and represent targets for investigation of cell death mechanisms in the mucinous subtype.

## Introduction

Mucinous colorectal cancer (CRC) is estimated to comprise approximately 10% of CRC cases and is diagnosed by histopathological identification of extracellular mucin comprising at least 50% of the tumor volume (1). Meta-analysis of 34 studies published in 2012 noted reported worse overall survival (OS) for patients with mucinous CRC compared to non-mucinous CRC (2). Inferior survival appears particularly related to rectal mucinous cancer with some recent studies noting no differences in colon cancer subgroups (3, 4). A meta-analysis of response to neoadjuvant chemoradiotherapy in mucinous and non-mucinous rectal cancers has identified lower rates of pathological complete response and tumor downstaging in mucinous cases (5).

Altered cell death and cell survival signaling contribute to chemotherapy and radiotherapy resistance in cancer cells. Mucin glycoproteins are associated with resistance to chemotherapy in both *in-vitro* and *in-vivo* models of CRC (6–9) (6–9). Mucinous CRC is associated with increased rates of *KRAS* and *BRAF* mutation (8). *BRAF* and *KRAS* are components of the RAS/MAPK pathway, and activation of this pathway promotes cell survival and reduces apoptosis (10).

Downstream of cell survival signaling, alterations in proteins responsible for executing cell death may also impair chemotherapy response (11). The programmed cell death mechanism, apoptosis is well described in colorectal cancer (12). Apoptosis is initiated by at least two pathways: extrinsically by death receptors and intrinsically by mitochondria-dependent pathways (13). The importance of Bcl-2 and its family members in cell death signaling has been demonstrated extensively in multiple cancers (14). BCL-2 proteins play a key role in the intrinsic mitochondria-dependent cell death pathway. Pro-apoptotic BCL2 family proteins include Bax, Bak and BID. Anti-apoptotic BCL2 family proteins include Bcl-2, Bcl-xl and Mcl-1. Activity of pro-apoptotic and anti-apoptotic BCL2 proteins controls mitochondrial outer membrane permeabilization and triggering of cytochrome c in response to intracellular stress (15). In the cytosol, cytochrome *c* binds to APAF1 and triggers its oligomerization. Caspase-9 is then recruited and activated by this platform, known as the apoptosome. Catalytically active caspase-9 cleaves and activates the executioner caspases-3 and -7 which mediate apoptosis through proteolytic cleavage of cellular proteins (15, 16).

The extrinsic apoptosis pathway is initiated by cell membrane proteins known as death receptors. Extrinsic apoptosis signaling is activated when ligands activate death receptors, recruiting FADD and initiator Caspase 8 to form the death inducing signaling complex (DISC). Activation of Caspase 8 at the DISC results in downstream activation of Caspase 3. (16). In addition, DISC formation leads to Caspase 8 mediated cleavage of BID which contributes to release of cytochrome *c* at the mitochondria (16).

In addition to apoptotic programmed cell death, necroptosis is an alternate programmed cell death mechanism described in many cancers, including CRC (12). Necroptosis is characterized by lysosomal membrane degradation, cytosol vacuolation, plasma membrane disintegration and cell lysis (17). Necroptosis is triggered by a number of stimuli, including tumor necrosis factor (TNF), Fas ligand signaling, and Toll-like receptors (18–20). Necroptosis is mediated by receptor interacting protein kinases (RIP) signaling, leading to phosphorylation of Mixed Lineage Kinase Domain Like Pseudokinase (MLKL) which proceeds to execute necroptotic events (17). Analysis of metastatic colorectal tumors by Lu et al. demonstrated higher MLKL in cancer responsive to FOLFOX chemotherapy (21). However, the prognostic role of MLKL in mucinous colorectal cancer remains to be clarified.

This study aimed to characterize apoptosis and necroptosis mediators in mucinous colorectal cancer to determine if differences in cell death signalling between mucinous and non-mucinous subtypes may represent a mechanism for chemotherapy resistance in mucinous colorectal cancer.

## Methods

### Cell Culture

SW1463, SW837, LS174T and HCT116 cells (ATCC-American Type Tissue Collection, LGC Standards, Middlesex, UK) were maintained in DMEM supplemented with 10% FBS, 2 mM L-glutamine, 100 U/ml penicillin, and 100 µg/ml streptomycin. Cell lines were verified at purchase by the ATCC or authenticated by STR profiling if in existing use in the laboratory. Cell lines were tested regularly by PCR to ensure they were not contaminated with mycoplasma. The SW1463 cell line is derived from a mucin producing and solid rectal adenocarcinoma in a 66 year old Caucasian female. The SW837 line is derived from a non-mucinous rectal adenocarcinoma in a 53 year old Caucasian male. The LS174T cell line is derived from a mucin producing colonic adenocarcinoma in a 58 year old Caucasian female. The HCT116 cells line is derived from a non-mucinous colonic adenocarcinoma in an adult male.

### Flow Cytometry-Based Detection of Cell Death Using PI

Cells were seeded at a density of 200,000 cells per well in a 12-well plate and allowed adhere overnight. Adherent cells were washed with PBS and treated with 25 μM 5-FU (Medac GmbH, Wedel, Germany). After 72h treatment, cells were detached using TrypLE Express (Thermo Fisher Scientific, Reading, UK) washed with Annexin-V binding buffer (10 mM HEPES, 140 mM NaCl, 2.5 mM CaCl2, pH 7.4) and stained with Annexin-V-FITC (1:200, BioLegend, London, UK). Stained cells were washed with Annexin-V binding buffer and stained with propidium iodide (1:500, BioLegend, London, UK). Stained cells were analyzed using a BD LSRII flow cytometer with the BD FACSDiva software (BD Biosciences, Oxford, UK) and Flowing Software (Turku Bioscience Centre, Turku, Finland). Doublet cells were excluded using an FSC-H and FSC-A scatter plot, and 10,000 single cells were measured per technical replicate. Cells staining positively with PI were considered to have undergone cell death. Annexin V staining was not employed for cell death quantification.

### Western Blotting

Cells were seeded in six-well plates at a density of 500,000 cells per well and allowed adhere overnight. Cells were treated with 5-FU (Medac GmbH, Wedel, Germany) for 72h. Proteins were isolated using RIPA buffer (140 mM NaCl, 10 mM Tris-HCl (pH 8.0), 1 mM EDTA, 1% Triton X-100, 0.1% sodium deoxycholate, 0.1% SDS) with freshly added protease, and phosphatase inhibitors (Sigma Aldrich Ireland Ltd, Dublin, Ireland). SDS-PAGE 12% gels were used for electrophoresis, and proteins were blotted onto nitrocellulose membrane using a TRIS-Glycine wet transfer method. Anti-Caspase-3, anti-Caspase-8, anti-Caspase-9, anti-pRIP1, anti-pMLKL, Anti-Bax, anti-Bak, anti-Mcl-1, anti- Bcl-xL, anti-BID antibodies were purchased (Cell Signaling Technology, MA, USA) and used at 1:1000 dilution. Anti-RIP1, anti-MLKL, anti-pRIP3, anti-Cleaved Caspase 8 antibodies (Cell Signaling Technology, MA, USA) were used at 1:500 dilution. Anti-RIP3, antibodies (Cell Signaling Technology, MA, USA) were used at 1:250 dilution. Anti-Bcl-2 antibodies were purchased (Santa Cruz BioTech Inc., Heidelberg, Germany) and used at 1:500 dilution. Antibody specifications are listed in a supplementary file (**Table S1**). Anti-actin antibody (1:2000, Sigma Aldrich Ireland Ltd, Dublin, Ireland) was used as a loading control. HRP-labeled anti-mouse and anti-rabbit antibodies (1:5000, Sigma Aldrich Ireland Ltd, Dublin, Ireland) were used for detection of the primary antibodies. Images were acquired using a Fuji LAS4000 imaging system (GE Healthcare, Buckinghamshire, UK) and chemiluminescence HRP substrate (Merck Millipore Immobilon™, Fisher Scientific Ireland Ltd, Dublin, Ireland). Measurement of protein band density was performed using ImageJ software (version 1.52A, Rasband, W.S., ImageJ, U. S. National Institutes of Health, Bethesda, Maryland, USA). Band density was expressed relative to the actin loading control for each replicate.

### Immunofluorescent Staining of Tumor Microarrays

Formalin-fixed, paraffin-embedded (FFPE) primary tumor tissue sections were obtained from patients with stage I-III rectal cancer (14 mucinous versus 39 non-mucinous) following tumor resection. Mucinous tumors were defined by a consultant histopathologist as those with greater than 50% of the tumor composed of mucin. Exposure to neo-adjuvant chemoradiotherapy prior to resection was recorded as a variable for analysis. Three tumor cores per patient were prepared on a microarray slide. Multiplexed immunofluorescence staining of the tumor micro-array was performed using the Cell DIVE™ technology (Cytiva, Marlborough, MA, USA). This involves multiple rounds of antibody staining performed on the same tissue section with mild dye oxidation between successive rounds of staining and imaging. Immunofluorescent images were processed to identify individual cells within the tumor section. Epithelial cells and stromal cells were segmented using antibody stains against DAPI, pan-cytokeratin and NaKATPase. Images and segmented cell data underwent a quality review process. Staining intensity was measured for epithelial cells within each tumor core and expressed as mean staining intensity at the patient level. Detailed description of the TMA image analysis technique is published in a larger analysis of 373 tumor cores by our research group (22). Institutional ethical approval was granted by the Beaumont Ethics (Medical Research) Committee. Tissue was provided from the Beaumont Hospital Colorectal Biobank with written consent provided by all patients.

### TCGA Gene Expression Analysis

A search was performed of The Cancer Genome Atlas (TCGA) to identify cases of CRC eligible for inclusion in the TCGA-COAD (Colon Adenocarcinoma) and TCGA-READ (Rectum Adenocarcinoma) datasets. Institutional approval was not required for open access data. Inclusion criteria specified Stage I-IV CRC, with histological subtypes of adenocarcinoma not otherwise specified (NOS) or mucinous adenocarcinoma. Other histological subtypes such as complex epithelial neoplasms and epithelial neoplasms NOS were excluded. Patients of both genders, all age groups and ethnicities were eligible for inclusion in the analysis. The demographic, pathological and clinical data for each eligible case were collated and harmonized from the GDC Legacy Archive and the TCGA-Clinical Data Resource publication (23). Gene expression data from primary tumor samples was extracted for each eligible case from the TCGA PanCanAtlas data-freeze release (24). Gene expression data was reported as level 4 batch-corrected and normalized mRNA expression derived from RNA-Seq quantification.

### Statistical Analysis

Variable frequencies were reported as means with standard deviation, medians with inter-quartile range or as percentages. The distribution of continuous variables was compared between groups using a Mann-Whitney U test for unpaired non-parametric variables and an unpaired t test for parametric variables. Categorical variable frequency was compared between groups using the Chi square test. A p value of 0.05 was defined as the cut-off for statistical significance. Data were analyzed using GraphPad PRISM 8.2.4 (GraphPad Software, CA, USA), IBM SPSS Statistics Version 25.0 (IBM Corp, NY, USA) and Python Version 3.8 (Python Software Foundation, Wilmington, DE, USA) software programs.

## Results

### Differential Expression of Genes Encoding Cell Death Signalling Proteins Observed in Mucinous and Non-Mucinous Colorectal Cancers in the TCGA Dataset.

As a preliminary investigation into cell death signalling in mucinous CRC, gene expression of key cell death signalling molecules was examined in the TCGA dataset, with findings compared between 74 mucinous and 524 non-mucinous colorectal cancers. Clinical and pathological characteristics of the included TCGA cohort are presented in **Supplemental Table 4**. We found that the median gene expression values for *Bak, Bcl-2, MCL1, RIP1* and *Caspase 3* were significantly higher in mucinous compared to non-mucinous cancers (p=0.011, p<0.001, p<0.001, p<0.001, p=0.025 respectively), while the median expression values for genes encoding *BID* and *Bcl-xL* were significantly lower in mucinous compared to non-mucinous cancers (p<0.001, p<0.001 respectively) (**Table 1**). When rectal cancers cases were examined independently from colon cancers cases in the TCGA, the median gene expression value for *MLKL* gene expression was higher in mucinous rectal cancers compared to non-mucinous rectal cancers, with a p value of 0.056. These findings suggested there may be differences between mucinous and non-mucinous CRC cell death signalling mechanisms.

**Table 1:** Gene expression of mucinous and non-mucinous colorectal cancer in TCGA dataset.

	Mucinous	Non-mucinous	p
	N=74	N=524	
Extrinsic apoptosis proteins			
CASP3	1199.3 (522.1)	1113.3 (494.9)	**0.025**
CASP8	804.9 (257.2)	823.8 (279.91	0.533
CASP9	294.4 (107.9)	286.7 (101.5)	0.084
Necroptosis proteins			
RIP1	984.6 (308.1)	813.5 (261.8)	**<0.001**
RIP3	427.8 (255.6)	453.5 (214.1)	0.402
MLKL	516.3 (200.1)	506.9 (213.1)	0.342
Intrinsic pro-apoptotic proteins			
BAX	1337.7 (738.6)	1297.1 (628.9)	0.805
BAK	1093.7 (528.5)	991.3 (575.2)	**0.011**
BID	867.3 (301.4)	1019.9 (492.8)	**<0.001**
Intrinsic anti-apoptotic proteins			
BCL-xl	2769.9 (1492.5)	4143.5 (2413.2)	**<0.001**
BCL-2	232.6 (177.9)	131.7 (137.9)	**<0.001**
MCL1	7474.4 (3300.4)	6029 (2911.4)	**<0.001**

Median (IQR), MWU.

Bold figures indicates p value <0.05.

### Higher Levels of Cleaved Caspase-3 are Observed in Mucinous Rectal Cancers.

**Figure 1:**
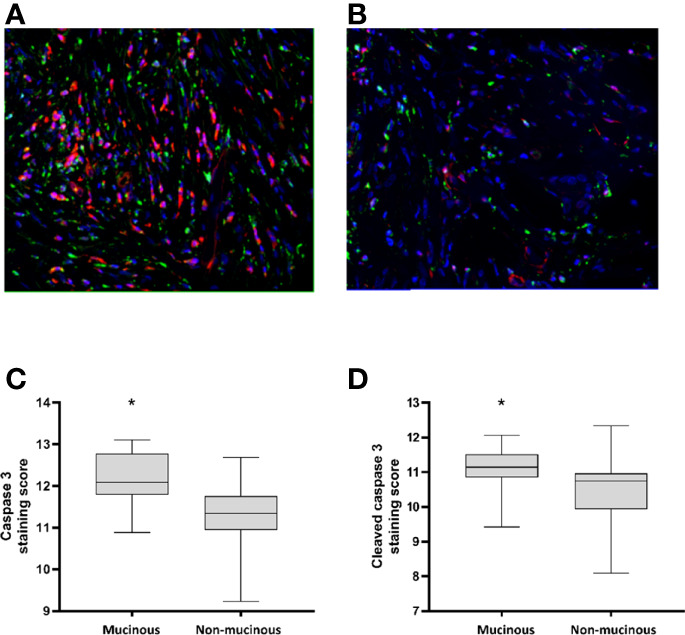
Caspase protein levels in mucinous versus non-mucinous colorectal cancer. **(A)** Mucinous rectal cancer tumor core stained with pro-Caspase 3 (red), cleaved-Caspase 3 (green), DAPI (blue=nucleus); **(B)** Non-mucinous rectal cancer tumor core stained with pro-Caspase 3 (red), cleaved-Caspase 3 (green), DAPI (blue=nucleus); **(C)** Quantification of pro-Caspase 3 protein level in 14 mucinous and 39 non-mucinous rectal cancers, *=p<0.05. p value for t-test comparing mean protein level between mucinous and non-mucinous cancers; **(D)** Quantification of cleaved-Caspase 3 protein level in 14 mucinous and 39 non-mucinous rectal cancers, *p<0.05. p value for t-test comparing mean protein level between mucinous and non-mucinous cancers.

In order to further explore this hypothesis, we investigated the expression levels of cell death signalling proteins in 14 resected mucinous and 39 resected non-mucinous rectal cancers. 69.2% of the cohort had received neoadjuvant CRT while 30.8% had proceeded directly to surgery. Multiplexed immunofluorescent detection of protein levels in a tumor microarray demonstrated higher mean immunofluorescence intensity of cleaved-Caspase 3 protein in mucinous rectal cancers compared to non-mucinous rectal cancers (p=0.027) (**Figure 1**). Additionally, higher mean immunofluorescence intensity of pro-Caspase 3 was also observed in mucinous rectal cancers compared to non-mucinous rectal cancers (p=0.003). While patients in both cohorts were exposed to neoadjuvant chemoradiotherapy prior to specimen sampling, the use of neoadjuvant chemoradiotherapy was less frequent in the mucinous cohort (57.1%) compared to the non-mucinous cohort (73.7%) (**Supplemental Table 2**). These findings indicate that a high rate of apoptotic signalling may occur in mucinous CRC at baseline. Necroptosis protein expression was also evaluated by measurement of MLKL protein levels in the mucinous and non-mucinous rectal tumors. However, levels of pMLKL were not available for measurement in the tissue microarray protocol, limiting evaluation of necroptosis signalling in resected tumours. Other prognostic variables including MSI status and use of neoadjuvant CRT did not show significant association with survival outcomes in the study cohort.

### Lower Levels of Bcl-2 Family Proteins in Mucinous vs Non-Mucinous Colorectal Cancer Cells

In order to further investigate necroptosis and apoptosis signalling in mucinous CRC we conducted a series of *in-vitro* experiments using a cell line model of mucinous and non-mucinous colorectal cancer. This preliminary study evaluated a small number of cell lines representing mucinous and non-mucinous cancer. Firstly, apoptosis and necroptosis signalling proteins were measured at baseline in mucinous and non-mucinous colon and rectal cancer cell lines.

**Figure 2:**
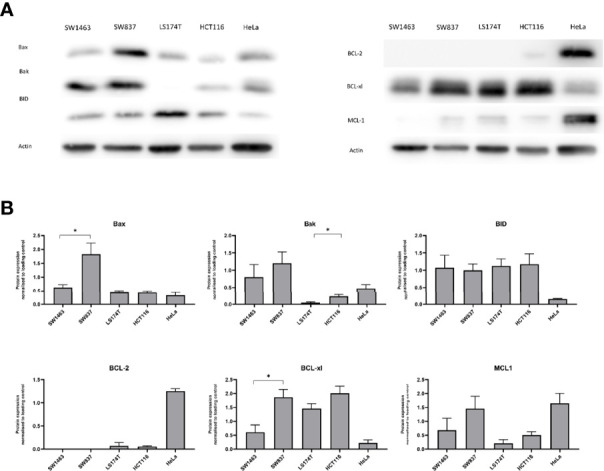
BCL-2 family protein levels in mucinous versus non-mucinous colon and rectal cancer cell lines. **(A)** Representative blots of BCL-2 family proteins at baseline; **(B)** BCL-2 family protein expression normalized to loading control. *p<0.05. p value for t test comparing mucinous versus non mucinous cell lines in colon and rectal cancer. N=3 experimental repeats.

Bcl-2 family proteins are upstream regulators of mitochondrial apoptosis. The levels of anti-apoptotic and pro-apoptotic Bcl-2 family proteins were compared in two mucinous and two non-mucinous colorectal cell lines at baseline (**Figure 2**). No significant difference in levels of the anti-apoptotic Bcl-2 protein was observed between mucinous and non-mucinous cell lines. However, the anti-apoptotic protein Bcl-xL showed lower levels in the mucinous rectal cell line compared to the non-mucinous rectal cell line. Protein levels of BCL-xl also tended to be lower in the mucinous colon cell line compared to the non-mucinous cell line.

The protein level of pro-apoptotic Bax was lower in the mucinous rectal cell line compared to the non-mucinous rectal cell line, while pro-apoptotic Bak protein levels were lower in the mucinous colon cell line compared to the non-mucinous colon cell line. BID protein levels were similar in mucinous vs. non-mucinous cell lines.

**Figure 3:**
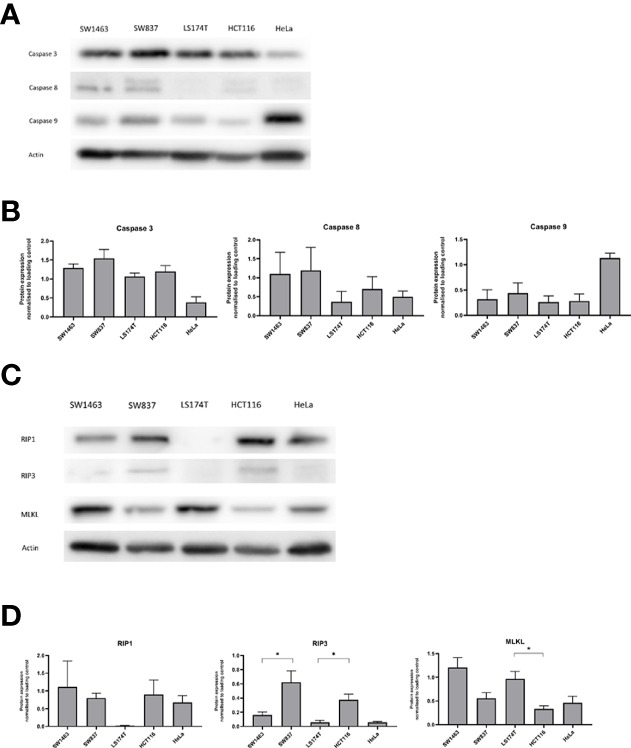
Pro-Caspase protein and Necroptosis protein levels in mucinous versus non-mucinous colon and rectal cancer cell lines. **(A)** Representative blots of pro-Caspase proteins at baseline; **(B)** Pro-Caspase protein expression normalized to loading control; **(C)** Representative blots of necroptosis proteins at baseline; **(D)** Necroptosis protein expression normalized to loading control. *p<0.05. p value for t test comparing mucinous versus non mucinous cell lines in colon and rectal cancer. N=3 experimental repeats.

We also investigated the protein levels of the initiator caspases of the mitochondrial (pro-Caspase-9) and extrinsic apoptosis pathway (pro-Caspase-8) in our mucinous and non-mucinous cell lines (**Figure 3**). No significant difference was observed in the protein levels of pro-Caspase 9 or pro-Caspase 8 between mucinous and non-mucinous colorectal cancer cell lines. Levels of pro-Caspase-3 were also not changed in our cell lines. Collectively, these observations suggested alterations particularly in the control of the mitochondrial apoptosis pathway by Bcl-2 family proteins in mucinous cancer cells.

### Altered Levels of Necroptosis Signaling Proteins in Mucinous Cancer Cells

We next sought to investigate the profile of necroptosis proteins in mucinous CRC. Necroptosis-executing MLKL protein levels were higher in mucinous colorectal cells compared to non-mucinous colorectal cells (**Figure 3**). Significantly higher protein levels of MLKL were noted in the mucinous colonic cell line compared to the non-mucinous colonic cell line (p=0.019). Higher protein levels of MLKL were likewise observed in the mucinous rectal cell line compared to the non-mucinous cell line (p=0.051). In contrast the protein levels of the upstream regulator of necroptosis RIP3 appeared lower in mucinous colorectal cells while. Significantly lower levels of RIP3 were noted in the mucinous rectal cell line compared to the non-mucinous rectal cell line (p=0.046). Significantly lower levels of RIP3 were also noted in the mucinous colonic cell line compared to the non-mucinous colonic cell line (p=0.019).

### Mucinous Tumor Cells Respond With Higher Levels of Necroptosis Signaling in Response to 5-FU

**Figure 4:**
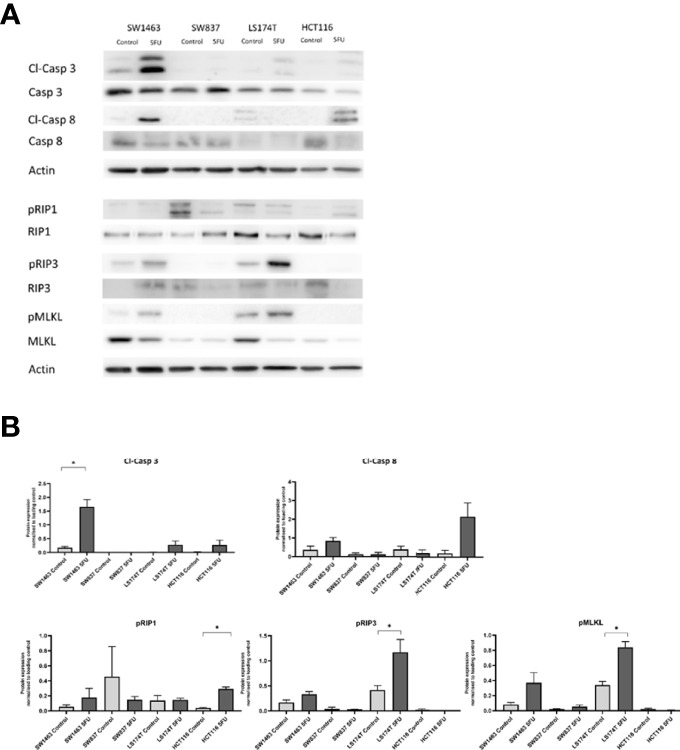
Apoptosis and necroptosis protein levels in response to 5-FU treatment in mucinous versus non-mucinous colon and rectal cancer cell lines. **(A)** Representative blots of apoptosis and necroptosis proteins in response to 5-FU; **(B)** Protein expression normalized to loading control. *p<0.05. p value for t test comparing mucinous versus non mucinous cell lines in colon and rectal cancer. N=3 experimental repeats.

We next aimed to investigate in more detail the activation of apoptosis and also necroptosis signaling pathways in mucinous and non-mucinous rectal and colon cell lines in response to 5-FU. We therefore measured cleaved-Caspase 8, cleaved-Caspase 3, phosphorylated RIP1, phosphorylated RIP3 and phosphorylated MLKL in mucinous and non-mucinous CRC cell lines treated with 25 μM 5-FU or DMSO control by western blot (**Figure 4** and **Supplemental Table 3**). A significant increase in cleaved-Caspase 3 was observed in SW1463 cells treated with 5-FU compared to control (p=0.004). Phosphorylated RIP1 was increased in the HCT116 cell line following 5-FU treatment compared to control (p=0.0007). Phosphorylated RIP3 was increased in the LS174T cell line following 5-FU treatment compared to control (p=0.048). Phosphorylated MLKL was increased in the LS174T cell line following 5-FU treatment compared to control (p=0.004). Collectively, these findings suggested a propensity for necroptotic signalling in mucinous CRC in response to 5-FU chemotherapy.

### Mucinous Colorectal Cancer Cells Have a Higher Basal Cell Death Rate When Compared to Non-Mucinous Colorectal Cancer Cells

**Figure 5:**
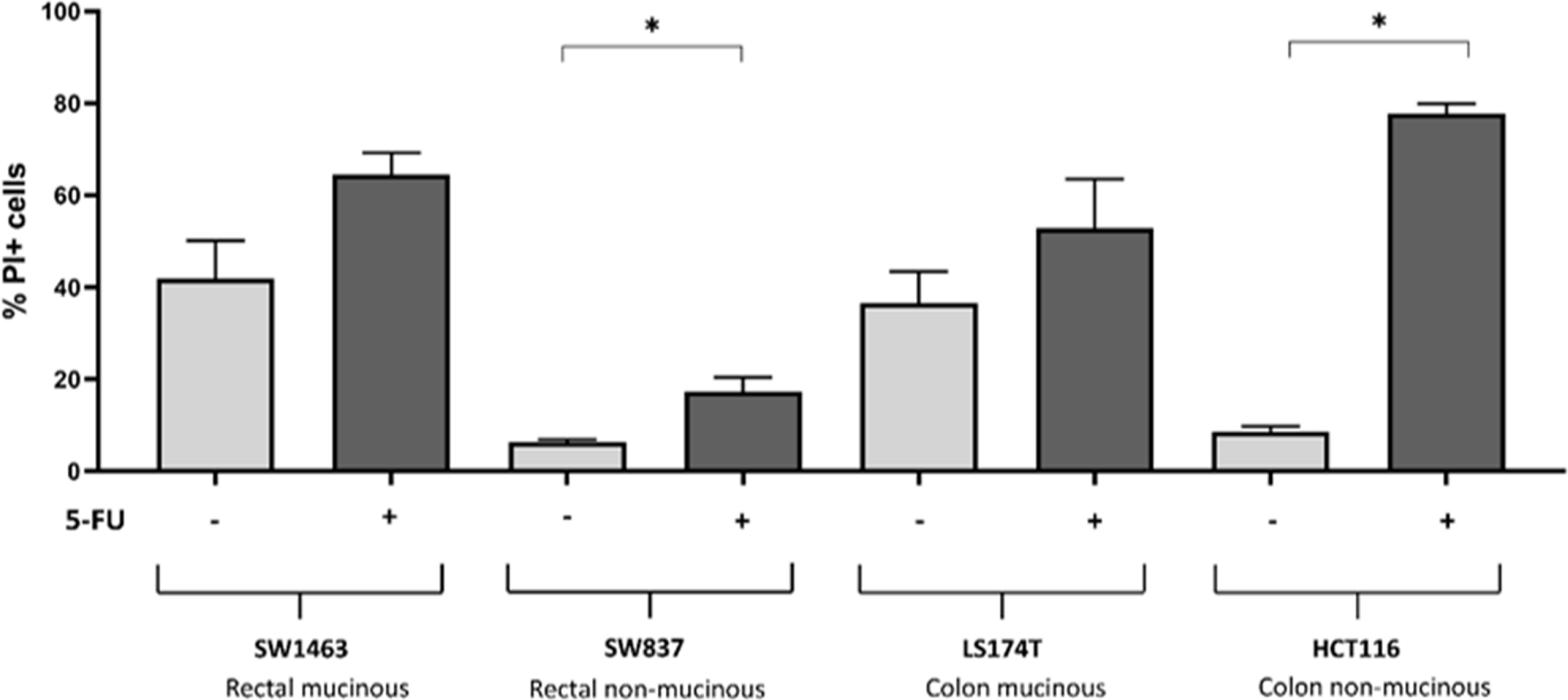
Cell death assay to determine cell death rate by measurement of PI positive cells. Cells treated with DMSO control versus 25μM 5-FU. * indicates p<0.05 for comparison between control and treated cells within cell line.

Flow cytometry measurement of propidium iodide stained cells was used to measure cell death in colon and rectal cancer cell lines under control conditions (0.1% DMSO as vehicle) and when exposed to 25 μM 5-FU (**Figure 5**). Of note, high basal cell death rate was observed in the mucinous rectal SW1463 cell line under control condition. No significant increase in cell death rate was observed in response to 5-FU treatment (p=0.079). Likewise, a high basal cell death rate was observed in the colonic mucinous LS174T cell line under control condition and but no significant increase in cell death rate was observed in response to 5-FU treatment (p=0.268). Low cell death rate was observed in the non-mucinous rectal cancer SW837 cell line under control condition. A small yet significant increase in cell death rate was observed in response to 5-FU treatment (p=0.026). Similarly, in the non-mucinous HCT116 colon cell line, low baseline cell death rate was observed, while a significant increase in cell death rate was observed with 5-FU treatment (p<0.001).

## Discussion

This analysis has identified significant differences in cell-death signaling proteins between mucinous and non-mucinous colorectal cancers. High cleaved-Caspase 3 expression was noted in mucinous tumors in a multiplex immunofluorescent analysis of tumor microarrays, a finding complimented by high cell death at baseline in *in-vitro* testing of mucinous cell lines. Significantly higher baseline expression of the executioner of necroptosis, MLKL was observed in mucinous cell lines. Treatment with 5-FU increased cell death in non-mucinous cell lines, while non-significant increases were observed in mucinous cell lines. Necroptosis pathways were preferentially activated in mucinous cell lines, suggesting that targeting necroptosis may enhance cell death in mucinous tumors.

Protein levels of Bax, Bak and Bcl-xL were lower in mucinous CRC cell lines. Dysregulation of Bcl-2 proteins occurs during tumor development and is associated with cellular resistance to chemotherapy treatments (25). Some prior immunohistochemical analyses have demonstrated no association between mucinous histology and Bcl-2 staining in CRC (26, 27). In contrast, Bhardwaj et al. identified Bcl-2 positivity more frequently in non-mucinous colorectal tumors compared to mucinous (28). We identified lower BCL-xL levels in mucinous CRC in mucinous cells *in-vitro*, and lower gene expression in mucinous tumors in the TCGA analysis. CRCs frequently show overexpression of Bcl-xL (29–31) suggesting it is likely that Bcl-xL protein levels are a more important determinant of cell survival in CRC. We observed high baseline death in mucinous cells *in-vitro* and high cleaved-Caspase 3 levels in resected rectal tumors, suggesting that Bcl-xL was unable to prevent the activation of pro-apoptotic Bak and Bax under baseline conditions, for example when cancerous cells are subjected to ‘physiological’ mitogenic, metabolic or environmental stress. Prediction of chemotherapy response on the basis of individual proteins levels is challenging given intra-tumoral heterogeneity and the complex regulation of BCL2 signaling. Prior work utilizing computational modelling to investigate the regulation of mitochondrial apoptosis has been able to successfully identify high-risk patients in CRC (32). It is anticipated that combination of cell-level protein measurement with computational modelling may facilitate better understanding of Bcl-2 regulation of apoptosis in mucinous CRC.

There is limited data exploring Caspase 3 expression in mucinous CRC. We here demonstrate increased levels of active caspase-3 in mucinous CRC. Previous work from this unit has explored the association of Caspase-3 with outcomes in colorectal cancer (33, 34). In Stage III CRC, the ability of cancer cells to activate Caspase-3 in response to chemotherapy, as determined by a quantitative analysis of all components of the mitochondrial apoptosis pathway in their inactive state, has been associated with good outcome in CRC (33, 34). However, high basal levels of active Caspase 3 prior to chemotherapy have also been shown to be associated with poorer outcome in CRC (35). In addition to its role as an executioner caspase, Caspase 3 also plays an important role in immunomodulation and tissue regeneration (36, 37). Although Caspase 3 activity causes cell death in the host cell, it may stimulate proliferation in neighboring non-apoptotic cells; the so-called “phoenix rising” phenomenon described by Li et al. (38, 39). Colorectal tumors are composed of heterogenous cell populations that vary in sensitivity to chemotherapy. Apoptotic cell death in a subpopulation of tumor cells may induce compensatory cell proliferation in resistant cancer cells, leading to tumor repopulation and chemotherapy resistance (35), a mechanism that may play an important role in the setting of mucinous CRC. Inhibition of caspase 3 mediated survival signaling, such as PGE2 or components of the Wnt signaling pathway, may represent a novel approach to reduce mucinous tumor repopulation following chemotherapy.

This analysis also identified altered expression of necroptosis mediators between mucinous and non-mucinous colorectal cancer. A small number of studies have suggested that MLKL expression may act as a prognostic biomarker in pancreatic and ovarian cancer (40, 41). Fauster et al. identified two existing anti-cancer agents, ponatinib and pazopanib, as inhibitors of necroptosis (42). However, novel agents to target necroptosis have not been developed for use in clinical practice. Analysis of metastatic colorectal tumors by Lu et al. demonstrated higher MLKL in cancer responsive to FOLFOX chemotherapy (21). We observed increased expression of MLKL at baseline and increased phosphorylation of MLKL in response to 5-FU in mucinous cell lines. We expected this protein profile would sensitize cells to death through necroptosis. However, cell death rates were modest when compared to the non-mucinous cell lines, a finding which could not be explained by the current data. We hypothesize that necroptosis signaling may have functions beyond cell death in colorectal cancer. Previous studies have highlighted that MLKL protein has multiple roles including immune cell interaction (43), inflammasome regulation, receptor/ligand internalization and exosome formation (44). Necroptosis is observed to provoke inflammation due the lytic nature of cell death (45). Inflammation arising from necroptosis can potentially stimulate tumor growth and metastasis (44). Seifert et al. have demonstrated that necroptotic signaling recruits inhibitory tumor associated macrophages and myeloid cells creating an immunosuppressive microenvironment with consequent tumor progression (46, 47). Immune and inflammatory signaling in response to necroptosis was not examined within the scope of this study. Future study of immune and inflammatory sequelae of necroptosis is suggested in mucinous colorectal cancer. Induction of immunogenic signaling by necroptosis may represent a therapeutic strategy by priming tumors for immune-mediated cytotoxicity.

This study acknowledges a number of limitations. Correlation of *in-vitro* protein levels with patient samples was limited by the availability of rectal cancer cases only for tumor microarray. In addition, pMLKL was not included in the multiplexed analysis. Limited numbers of mucinous tumors were available for inclusion as rectal mucinous cancer occurs less frequently than mucinous colon cancer (10). *In-vitro* analysis of cancer cell lines is a valuable tool to investigate molecular features of tumor response to chemotherapy. However, immortalized cell lines cannot wholly replicate tumor heterogeneity or cell signaling within a tumor microenvironment. Tumor side was not examined as a variable in the *in-vitro* aspect of this study. Given the differences between rectal and right sided colon cancers we did not directly compare these cell lines. Comparison was conducted between the mucinous and non-mucinous subtype within the rectal and colon groups. Additionally, high variability in protein expression may be observed between different cell lines from the same type of cancer. The number of cell lines included in this work was limited due to limited numbers of available mucinous cell lines but future work in this area is recommended to include a large panel of mucinous and non-mucinous colorectal cancer cell lines. Annexin/PI staining is a standard laboratory protocol for flow cytometry. PI positive stained cells were selected to identify cells undergoing cell death in this study. We did not possess a stain to determine cells undergoing necroptosis and thus could not quantify apoptosis versus necroptosis as the mechanism of cell death in this experiment. We suggest future experiment with appropriate necroptosis markers in addition to Annexin to determine the mechanism of cell death by flow cytometry. Comparison of gene expression data from the TCGA dataset with protein expression data in patient samples and cell lines is limited by the observation that gene expression may not directly predict protein expression due to post-transcriptional alteration of protein action.

In conclusion, we here show that molecular differences between mucinous and non-mucinous colorectal cancers extend to cell death signaling with differences observed in the expression of apoptotic and necroptotic mediators. Mucinous CRC demonstrated lower Bcl-xL expression, elevated basal caspase 3 activation, and lack of a cell death response to 5-FU treatment despite increased necroptosis signaling. An interesting preliminary finding from this research is that MLKL expression appears increased in mucinous cell lines at baseline and pMLKL expression was elevated in response to 5-FU. These finding may inform several strands of therapeutic investigation in mucinous CRC including caspase inhibition to augment necroptosis induction, direct necroptosis molecule targeting and priming of mucinous tumors for immune-mediated cytotoxicity by induction of necroptotic cell death.

## Data Availability Statement

Publicly available datasets were analyzed in this study. This data can be found here: Supplemental Data RNA - EBPlusPlusAdjustPANCAN_IlluminaHiSeq_RNASeqV2.geneExp.Tsv which is accessible from https://gdc.cancer.gov/about-data/publications/pancanatlas. Data is provided by the National Institute of Health’s National Cancer Institute through its Genomic Data Commons.

## Ethics Statement

The studies involving human participants were reviewed and approved by the Beaumont Ethics (Medical Research) Committee. The patients/participants provided their written informed consent to participate in this study.

## Author Contributions

Study design: JB and JP; Data collection: EO’C, IR, MS, AL, and OB; TMA preparation: IR, OB, and TO’G; TMA analysis: EM, SC, and FG; *In-vitro* experimentation: EO’C, IR, and JP; Data analysis: EO’C, MS, AL, SC, DL, FG, JB, and JP; Manuscript preparation: EO’C, FG, DL, DM, JB, and JP. All authors contributed to the article and approved the submitted version.

## Funding

This work was funded by a US-Northern Ireland-Ireland Tripartite grant from Science Foundation Ireland and the Health Research Board to JHMP (16/US/3301) and the National Cancer Institute (Systems Modeling of Tumor Heterogeneity and Therapy Response in Colorectal Cancer; to FG). DBL was supported by US-Ireland R01 award (NI Partner supported by HSCNI, STL/5715/15). EO’C is support by the Bons Secours Hospital Dublin through an RCSI StAR MD scholarship and the Beaumont Hospital Cancer Research Trust.

## Conflict of Interest

Authors SC, EM and FG were employed by company GE Global Research.

The remaining authors declare that the research was conducted in the absence of any commercial or financial relationships that could be construed as a potential conflict of interest.

## Publisher’s Note

All claims expressed in this article are solely those of the authors and do not necessarily represent those of their affiliated organizations, or those of the publisher, the editors and the reviewers. Any product that may be evaluated in this article, or claim that may be made by its manufacturer, is not guaranteed or endorsed by the publisher.
