# Supplementary material for: Apoptotic and Necroptotic Mediators are Differentially Expressed in Mucinous and Non-Mucinous Colorectal Cancer
Source: Front Oncol. 2022 Jul 14;12:815001. doi: 10.3389/fonc.2022.815001 (PMC9334008; doi:10.3389/fonc.2022.815001)
Supplement: Supplementary file 1 [file DataSheet_1.pdf]

Supplemental data

Table S1. Antibody specifications.

Protein target	Size (kDA)	Secondary	Dilution	Company	Purchase Code
Caspase 3	35	Rabbit	1:1000	Cell Signaling Technology	#9662
Caspase 8	57	Mouse	1:000	Cell Signaling Technology	#9746
Caspase 9	47	Rabbit	1:1000	Cell Signaling Technology	#9502
Cleaved Caspase 3	17,19	Rabbit	1:250	Cell Signaling Technology	#9664
Cleaved Caspase 8	43,44	Rabbit	1:500	Cell Signaling Technology	#9496
BCL-2	26	Mouse	1:500	Santa Cruz	#sc-509
BCL-xl	20	Rabbit	1:1000	Cell Signaling Technology	#2764
Bax	25	Rabbit	1:1000	Cell Signaling Technology	#2772
Bak	25	Rabbit	1:1000	Cell Signaling Technology	#12105
BID	22	Mouse	1:1000	Cell Signaling Technology	#2002
MCL1	40	Rabbit	1:1000	Cell Signaling Technology	#5453
RIP1	78	Rabbit	1:500	Cell Signaling Technology	#44590
RIP3	46-62	Rabbit	1:250	Cell Signaling Technology	#95702
MLKL	54	Rabbit	1:500	Cell Signaling Technology	#91689
p-RIP1	78-82	Rabbit	1:1000	Cell Signaling Technology	#65746
p-RIP3	46-62	Rabbit	1:500	Cell Signaling Technology	#93654
p-MLKL	54	Rabbit	1:1000	Cell Signaling Technology	#91689

Table S2. Characteristics of mucinous and non-mucinous rectal cancer cases in tumor micro-array

	**Full Cohort**	**Mucinous**	**Non-mucinous**	**p**
	N=53	N=14	N=39	
Age (years)	68.25 (11.5)	63.5 (16.3)	69.95 (8.87)	0.070
Sex (male)	30 (56.6%)	7 (50.0%)	23 (59.0%)	0.756
AJCC Stage				0.503
I	7 (13.2%)	2 (14.3%)	5 (12.8%)	
II	24 (45.3%)	8 (57.1%)	16 (41.0%)	
III	22 (41.5%)	4 (28.6%)	18 (46.2%)	
T Stage				0.620
T1	2 (3.8%)	0 (0.0%)	2 (5.1%)	
T2	11 (20.8%)	3 (21.4%)	8 (20.5%)	
T3	35 (62.3%)	8 (57.1%)	27 (64.1%)	
T4	7 (13.2%)	3 (21.4%)	4 (10.3%)	
Node status				0.061
N0	31 (58.5%)	10 (71.4%)	21 (53.8%)	
N1	16 (30.2%)	1 (7.1%)	15 (38.5%)	
N2	6 (11.3%)	3 (21.4%)	3 (7.7%)	
Grade (54)				0.556
Well	1 (1.9%)	0 (0.0%)	1 (2.6%)	
Moderate	49 (94.2%)	14 (100%)	35 (92.1%)	
Poor	2 (3.8%)	0 (0.0%)	2 (5.3%)	
R0 margins	51 (96.2%)	12 (85.7%)	39 (100%)	0.066
LVI	9 (17.0%)	1 (7.1%)	8 (20.5%)	0.416
PNI	7 (13.2%)	2 (14.3%)	5 (12.8%)	1.000
EMVI	9 (17.0%)	1 (7.1%)	8 (20.5%)	0.416
MSI	3 (5.8%)	2 (14.3%)	1 (2.6%)	0.173
KRAS mutant	12 (22.6%)	5 (35.7%)	7 (17.9%)	0.263
BRAF mutant	2 (3.8%)	1 (7.7%)	1 (2.6%)	0.441
Neoadjuvant chemoradiotherapy (52)	36 (69.2%)	8 (57.1%)	28 (73.7%)	0.316
Adjuvant chemotherapy (47)	24 (51.1%)	8 (66.7%)	15 (42.9%)	0.193
Recurrence	7 (13.2%)	2 (14.3%)	5 (12.8%)	1.000
Cancer-related mortality	1 (1.9%)	1 (7.1%)	0 (0.0%)	0.264
Overall mortality	17 (32.1%)	3 (21.4%)	15 (35.9%)	0.506

N (%) or mean (SD), unpaired t test to compare continuous variables. Chi square to compare categorical variables.

Table S3(a) Mean protein expression in mucinous and non-mucinous rectal cancer cell lines treated with 25uM 5FU

	Mucinous (SW1463)			Non-mucinous (SW837)		
	Control	25uM 5FU	P	Control	25uM 5FU	p
CC3	0.17 (0.07)	1.66 (0.44)	**0.004**	0.005 (0.008)	0.006 (0.01)	0.972
CC3/C3	0.19 (0.008)	1.55 (0.10)	**<0.001**	0.006 (0.009)	0.005 (0.009)	0.985
CC8	0.36 (0.34)	0.84 (0.31)	0.146	0.12 (0.12)	0.12 (0.15)	0.998
CC8/Casp8	0.51 (0.43)	1.53 (1.48)	0.448	0.14 (0.07)	0.18 (0.05)	0.633
pRIP1	0.05 (0.04)	0.18 (0.21)	0.378	0.45 (0.69)	0.15 (0.07)	0.487
pRIP1/RIP1	0.53 (0.33)	0.54 (0.36)	0.995	1.73 (2.63)	0.34 (0.15)	0.413
pRIP3	0.16 (0.09)	0.33 (0.09)	0.097	0.04 (0.06)	0.03 (0.01)	0.807
pRIP3/RIP3	1.44 (0.57)	9.47 (8.03)	0.272	0.05 (0.08)	0.20 (0.08)	0.112
pMLKL	0.08 (0.05)	0.36 (0.23)	0.104	0.02 (0.01)	0.05 (0.04)	0.238
pMLKL/MLKL	0.08 (0.04)	0.62 (0.09)	**0.001**	0.07 (0.05)	0.17 (0.05)	0.083

Table S3(b) Mean protein expression in mucinous and non-mucinous colon cancer cell lines treated with 25uM 5FU

	Mucinous (LS174T)			Non-mucinous (HCT116)		
	Control	25uM 5FU	P	Control	25uM 5FU	p
CC3	0.006 (0.01)	0.27 (0.24)	0.122	0.01 (0.01)	0.27 (0.30)	0.218
CC3/C3	0.01 (0.01)	0.32 (0.01)	**<0.001**	0.02 (0.03)	0.40 (0.02)	**0.0001**
CC8	0.38 (0.29)	0.21 (0.31)	0.552	0.20 (0.28)	2.16 (1.29)	0.062
CC8/Casp8	2.64 (1.58)	1.50 (1.62)	0.549	0.48 (0.54)	6.17 (1.46)	**0.035**
pRIP1	0.13 (0.11)	0.14 (0.04)	0.904	0.04 (0.01)	0.29 (0.04)	**0.0007**
pRIP1/RIP1	0.23 (0.18)	0.33 (0.09)	0.435	0.04 (0.01)	0.63 (0.24)	**0.013**
pRIP3	0.41 (0.15)	1.17 (0.44)	**0.048**	0.02 (0.03)	0.01 (0.002)	0.441
pRIP3/RIP3	1.63 (0.84)	3.81 (2.03)	0.160	0.02 (0.04)	0.03 (0.02)	0.737
pMLKL	0.33 (0.08)	0.84 (0.12)	**0.004**	0.02 (0.02)	0.01 (0.009)	0.446
pMLKL/MLKL	0.45 (0.25)	3.72 (2.45)	0.083	0.12 (0.18)	0.03 (0.06)	0.496

Table S4. Characteristics of 598 mucinous and non-mucinous colorectal cases in TCGA dataset

	Full Cohort	Mucinous	Non-mucinous	p
	N=598	N=74	N=524	
Age (years)	67.5 (18)	66.5 (25.5)	68 (18)	0.409
Sex (male)	318 (53.2%)	37 (50.0%)	281 (53.6%)	0.619
Cancer type				0.116
Colon	434 (74.4%)	60 (82.2%)	374 (73.3%)	
Rectal	149 (25.6%)	13 (17.8%)	136 (26.7%)	
Site (583)				<0.001
Proximal	265 (45.5%)	50 (68.5%)	215 (42.2%)	
Distal	169 (29.0%)	10 (13.7%)	159 (31.2%)	
Rectal	149 (25.6%)	13 (17.8%)	136 (26.7%)	
AJCC Stage (579)				0.542
I	100 (17.3%)	10 (13.7%)	90 (17.8%)	
II	222 (38.3%)	30 (41.1%)	192 (37.9%)	
III	172 (29.7%)	25 (34.2%)	147 (29.1%)	
IV	85 (14.7%)	8 (11.0%)	77 (15.2%)	
T Stage (577)				0.139
T1-T2	101 (17.5%)	9 (12.5%)	92 (18.2%)	
T3	408 (70.7%)	50 (69.4%)	358 (70.9%)	
T4	68 (11.8%)	13 (18.1%)	55 (10.9%)	
Node status				0.758
N0	341 (57.3%)	41 (55.4%)	300 (57.6%)	
N1	144 (24.2%)	17 (23.0%)	127 (24.4%)	
N2	110 (18.5%)	16 (21.6%)	94 (18.0%)	
LVI (536)	221 (41.2%)	24 (35.8%)	197 (42.0%)	0.356
EMVI (515)	128 (24.9%)	13 (21.3%)	115 (25.3%)	0.636
MSI (561)	90 (16.0%)	22 (31.9%)	68 (13.8%)	<0.001
Progression (598)	152 (25.4%)	18 (24.3%)	134 (25.6%)	0.887
Cancer-related mortality (578)	75 (13%)	10 (14.3%)	65 (12.8%)	0.706
Overall mortality (598)	121 (20.2%)	18 (24.3%)	103 (19.7%)	0.355

N (%). P value for X2 test. Numbers in brackets indicate variables with incomplete dataset.
